# The clinical significance of the incorporation of tissue Doppler imaging into low-dose Dobutamine stress echocardiography in patients with aortic stenosis prior to Transcatheter aortic valve implantation

**DOI:** 10.1186/s12872-020-01700-0

**Published:** 2020-09-14

**Authors:** Sahrai Saeed, Joerg Kellermair, Jon Herstad, Øyvind Bleie

**Affiliations:** 1 Department of Heart Disease, Haukeland University Hospital, Jonas Lies vei 65, 5021 Bergen, Norway; 2 Department of Cardiology, Kepler University Hospital, Medical Faculty Johannes Kepler University Linz, Krankenhausstrasse 9, 4020 Linz, Austria

**Keywords:** Aortic stenosis, Dobutamine-stress-echocardiography, Tissue Doppler, Mitral annular systolic velocity, Intrinsic contractile function

## Abstract

**Background:**

Low-dose dobutamine stress echocardiography (DSE) is indicated in patients with low flow (stroke volume index [SVi] < 35 ml/m^2^) low gradient (mean pressure gradient < 40 mmHg) and left ventricular ejection fraction (LVEF) < 50% aortic stenosis (AS) to assess LV contractile reserve (> 20% increase in SVi) and severity grade of AS. Severe AS is defined by a mean pressure gradient of 40 mmHg occurring at any time during the test when aortic valve area remains < 1.0 cm^2^.

**Case presentation:**

This case report highlights the utility of mitral annular systolic velocity (S′) by tissue Doppler imaging and peak LV outflow tract (LVOT) velocity as markers of LV intrinsic contractile function during DSE in a patient with low flow low gradient AS and reduced EF prior to transcatheter aortic valve implantation (TAVI).

**Conclusions:**

Mitral annular S′ and peak LVOT velocities are reliable markers of LV intrinsic contractile function and should be incorporated into routine low-dose DSE.

## Background

This case is of clinical interest for physicians and sonographers who perform DSE in patient with classical low flow low gradient AS to assess contractile reserve, the patterns of flow normalization and the hemodynamic severity of AS. However, the incorporation of mitral annular S′ and peak LVOT velocity into low dose DSE may provide additional information on the LV intrinsic contractile function and the potentials for recovery of LV function following valve intervention.

## Case presentation

A 82-year-old male with known coronary artery disease (previous coronary artery bypass grafting following myocardial infarction), diabetes type II, hypertension and hypercholesterolemia, and smoking-induced chronic obstructive pulmonary disease (FEV1 69%) was referred to our heart valve clinic for assessment of AS. An echocardiogram 2 years earlier had shown LVEF of 60% and a moderate AS. He had experienced progressive dyspnea over the past 4 weeks (NYHA function class III).

His medications included vildagliptin 50 mg twice daily, empagliflozin 25 mg once daily (OD), liraglutid 1.2 mg s.c. OD, aclidinium bromide/formoterol fumarate dehydrate inhalation 340/12 μg twice daily, aspirin 75 mg OD, metoprolol depot 50 mg OD and atorvastatin 40 mg OD.

His body surface area was 1.80 m^2^. On physical examination, there was a systolic murmur, grade 4/6 on the right and left 2nd intercostal space, with the preserved second heart sound. His blood pressure was 124/79 mmHg.

### Investigations

Laboratory tests: Apart from non-fasting serum glucose (11.3 mmol/L) and NT-pro-BNP (260 ng/L), all other blood tests were normal including troponin T (9 ng/L), creatinine (67 μmol/L), eGFR (67 mL/min/1.73m^2^), sodium (139 mmol/L) and potassium (4.3 mmol/L).

Chest X-ray was unremarkable.

Coronary angiography showed a normal left main stem, native three-vessel disease, but open coronary bypass grafts (left internal mammillary artery to left anterior descending artery, and saphenous vein grafts to circumferential artery and right coronary artery). No new obstructive lesions were revealed. Heart CT showed a tricuspid aortic valve with Ca score of 2140 Agatston units.

An ECG showed sinus rhythm with heart rate of 58 beat per minute, and a Q-wave in leads II, III and AVF.

### Echocardiography

**Fig. 1:**
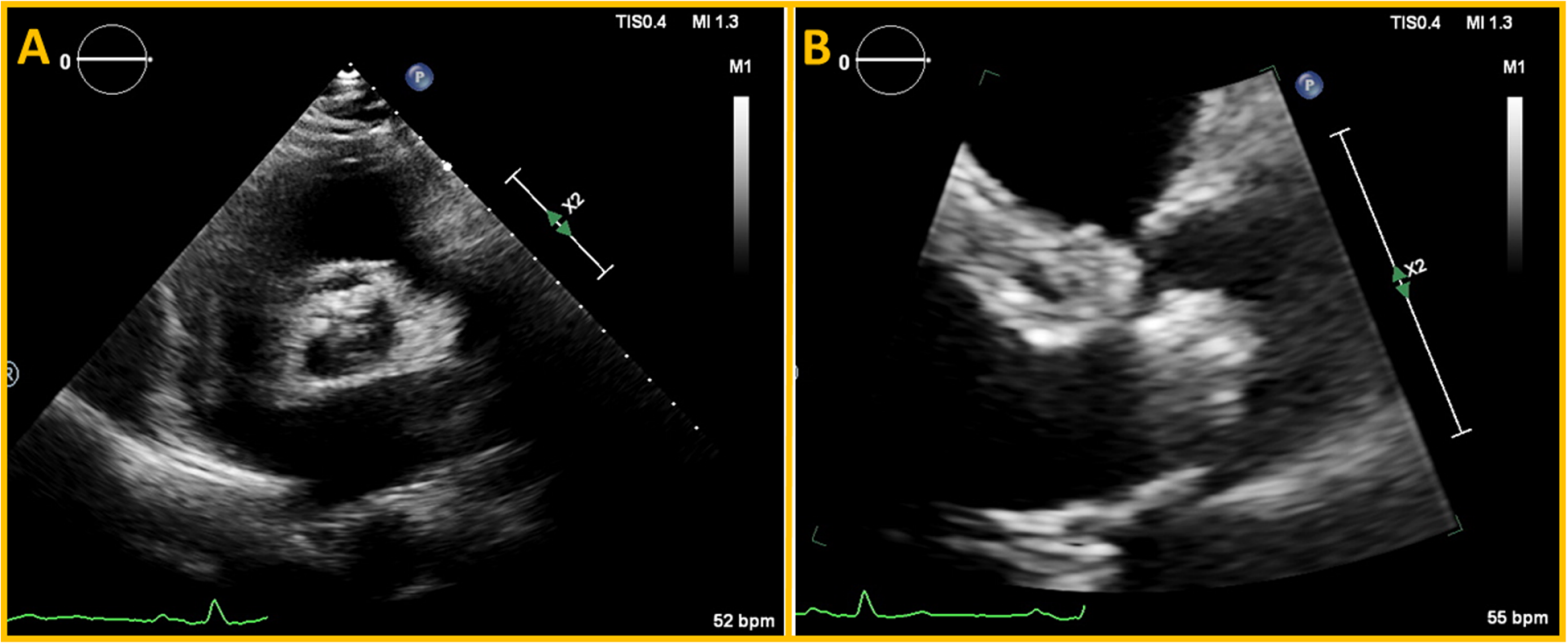
Aortic valve calcification in parasternal short axis (**a**) and long axis views (**b**)

**Fig. 2:**
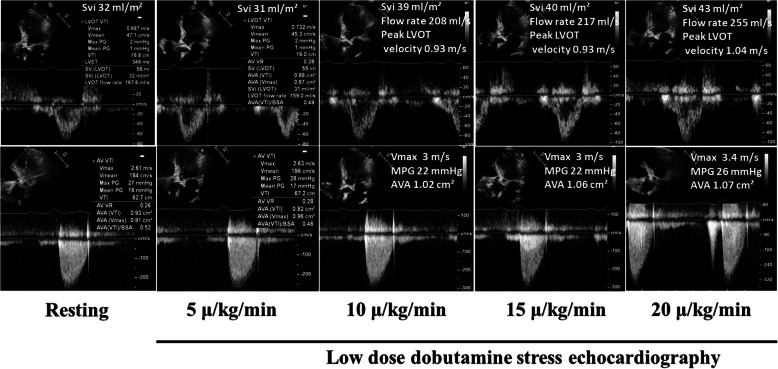
Changes in aortic flow, peak LVOT velocity, MPG, Vmax and AVA from rest to peak dobutamine stress echocardiography. AVA, aortic valve area; LVOT, left ventricular outflow tract; MPG, mean aortic pressure gradient; SVi, stroke volume index; Vmax, peak aortic jet velocity

**Fig. 3:**
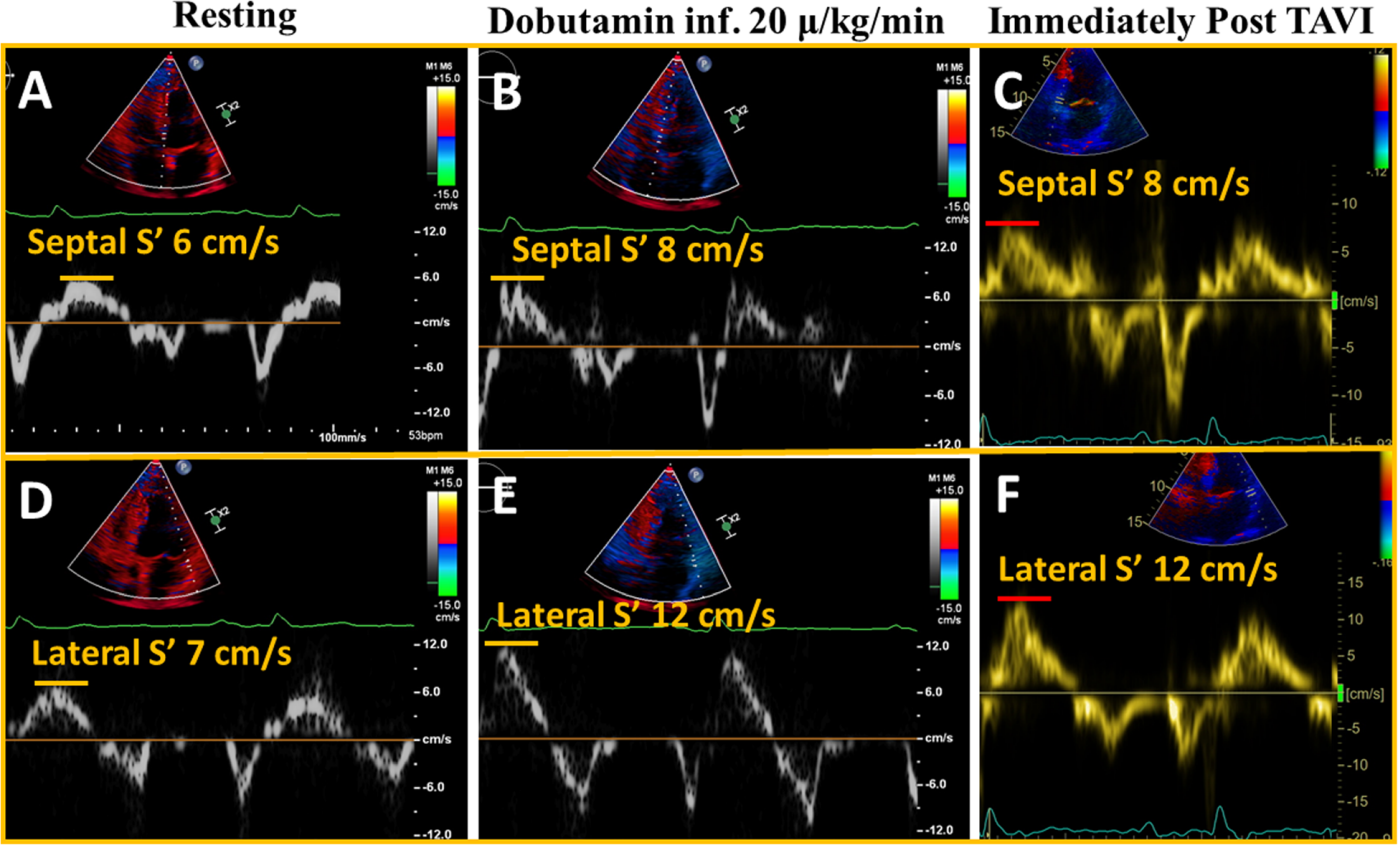
Septal and lateral S′ at rest (**a**, **d**), peak dobutamine stress echocardiography (**b**, **e**) and immediately after TAVI (**c**, **f**)

Conventional echocardiography (Philips “Epiq 7”; Philips Medical Systems, Bothell, WA) showed reduced LVEF (42%) and hypokinesia in the ventricular septum and inferior wall following previous myocardial infarction. The Aortic valve was heavily calcified (Fig. 1). Flow rate was 168 ml/s and stroke volume index (SVi) 32 ml/m^2^. AS was mild by peak aortic jet-velocity (2.6 m/s), but severe by aortic valve area (0.9 cm^2^). A mild aortic and mitral regurgitations were noted. There was no sign of right-sided valvular heart disease or pulmonary hypertension. A low-dose DSE (3 min stages with a starting dose 5 μg/kg/min increasing to 10, 15 and 20 μg/kg/min) was indicated to assess contractile reserves and the severity grade of AS, i.e. differentiate true severe from pseudo-severe [1]. The main results of DSE are reported in Table 1. Briefly, SVi increased from 32 ml/m^2^ to 43 ml/m^2^ (34% increase), aortic jet velocity from 2.6 m/s to 3.4 m/s and aortic valve area from 0.93 cm^2^ to 1.07 cm^2^. LVEF increased from 42 to 50%, peak LVOT velocity from 0.69 m/s to 1.04 m/s (51% increase), septal mitral annular S′ from 6 cm/s to 8 cm/s (33% increase) and lateral mitral annular S′ from 7 cm/s to 12 cm/s (71% increase) (Table 1 and Figs. 2 and 3).

**Table 1:** Measures of resting and low-dose dobutamine stress echocardiography in a patient with low flow, low gradient, low EF aortic stenosis

Echocardiographic parameters	Resting	Stage I 5 μ/kg/min	Stage II 10 μ/kg/min	Stage III 15 μ/kg/min	Stage IV 20 μ/kg/min
Heart rate (beat per minute)	52	54	53	55	60
Peak aortic jet velocity (m/s)	2.6	2.6	3.0	3.0	3.4
Mean pressure gradient (mmHg)	16	17	22	22	26
Peak LVOT velocity (m/s)	0.69	0.73	0.93	0.93	1.04
Aortic valve area (cm^2^)	0.93	0.82	1.02	1.06	1.07
Stroke volume index (ml/m^2^)	32	31	39	40	43
Systolic ejection time (ms)	346	343	322	323	300
Flow rate (ml/s)	168	159	208	217	255
LV ejection fraction (%)	42	NA	NA	NA	50
Septal S′ (cm/s)	6	NA	NA	NA	8
Lateral annular S′ (cm/s)	7	NA	NA	NA	12

Left ventricular outflow track (LVOT) diameter 2.1 cm

### Treatment and outcome

**Fig. 4:**
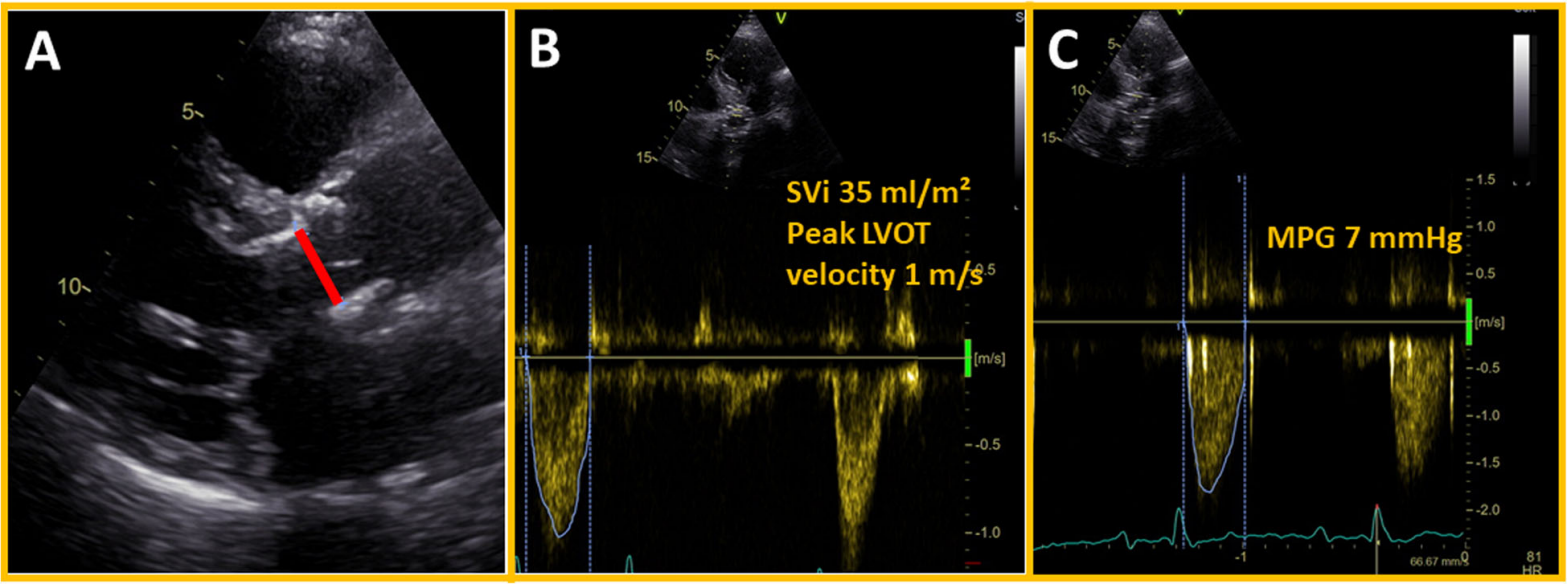
Bioprosthesis in aortic position and annulus diameter (**a**), SVi and peak LVOT velocity (**b**) and MPG immediately after TAVI (C). LVOT, left ventricular outflow tract; MPG, mean aortic pressure gradient; SVi, stroke volume index

This patient with AS, LV dysfunction and coronary artery disease who appeared to be increasingly symptomatic with NYHA function class III for four weeks prior to the admission, underwent a coronary angiography which did not reveal any new flow-limiting lesions requiring percutaneous coronary intervention or explaining the increase in symptoms. A low-dose DSE revealed adequate contractile reserves and a moderate to severe AS, supported by a heavily calcified aortic valve as seen with heart CT. His symptoms and LV dysfunction were primarily attributed to AS. His medical treatment was optimised by adding an angiotensin-converting enzyme inhibitor. After discussion at the heart team, he was considered eligible for TAVI, and a SAPIEN 3 Ultra® 26 mm was successfully implanted transfemoraly 4 weeks after DSE without any complications. An echocardiogram at discharge showed decrease in mean aortic pressure gradient to 7 mmHg, increase in SVi to 35 ml/m^2^, peak LVOT velocity to 1 m/s (Fig. 4), and LVEF to 50%. Septal S′ increased to 8 cm/s and lateral S′ to 12 cm/s (Fig. 3).

### Follow-up at 6-weeks following TAVI

Patient’s symptoms gradually improved within few weeks after TAVI. At 6 weeks follow-up he remained free of symptoms and systolic tissue Doppler velocities and peak LVOT velocity remained stable (the same values as at discharge). SVi further increased to 55 ml/m^2^ and peak LVOT velocity to 1.27 m/s.

## Discussion and conclusion

In routine clinical practice, a low dose DSE is essential for subcategorization of classical low flow low gradient AS into true severe and pseudo-severe, and assessment of contractile reserves, reflected by > 20% increase in SVi. Low dose DSE is a relatively safe and tolerable investigation, and provides valuable information on LV contractile reserve, which has important implications in terms of surgical risk stratification [2, 3]. During DSE several types of hemodynamic responses may be observed (Table 2) [1]. In our patient, a type B response was observed, suggesting that AS was per definition moderate (Tables 1 and 2). However, the aortic valve area at peak stress was 1.07 cm^2^, a borderline cut-off between moderate and severe. Aortic valve was heavily calcified with a Ca score of 2140 Agatston units by CT, indicating a hemodynamically severe AS. In order to restore LV function and treat symptoms, the removal of valvular resistance/stenosis by TAVI was essential. It is known that the prognosis of moderate AS is no longer benign [4]. In a recent large study of AS patients from Australia, the authors demonstrated that moderate and severe AS had equally high rates of mortality when left untreated [5]. We have recently shown that patients with moderate and severe AS had similar degree of aortic damage, as reflected by carotid-femoral pulse wave velocity [6].

**Table 2:** Types of hemodynamic response during low-dose DSE in patients with low flow (stroke volume index < 35 ml/m^2^), low gradient (mean pressure gradient < 40 mmHg) and low left ventricular ejection fraction (< 50%) severe aortic stenosis (aortic valve area < 1.0 cm^2^)

	Stroke volume and ejection fraction	Mean pressure gradient	Aortic valve area	Interpretation
**A**	**↑**	**↑**	**↔**	Severe AS
**B**	**↑**	**↔**	**↑**	Moderate AS
**C**	**↔**	**↔**	**↔**	Severe AS, severe LV dysfunction

*AS* aortic stenosis, *LV* left ventricular, *DSE* dobutamine stress echocardiography

LVEF and SVi are the conventional markers of heart pump function and may not necessarily reflect the true intrinsic contractile function of the LV. This case report illustrates that besides the traditional contractile reserves as assessed by > 20% increase in SVi, other routine echocardiographic parameters such as mitral annular S′ by tissue Doppler and peak LVOT velocity may also provide important clinical information on the recovery of LV function following valve intervention. During DSE, the septal S′ increased by 33% to 8 cm/s, lateral annular S′ by 71% to 12 cm/s, and peak LVOT velocity increased by 51% to 1.04 m/s (Figs. 2 and 3). The same level of LV performance achieved by DSE was seen at rest immediate after TAVI. For comparison, SVi increased by 34% to 43 ml/m^2^ during DSE pre TAVI, remained at 35 ml/m^2^ post TAVI, but further increased to 55 ml/m^2^ 6 weeks after TAVI. In a previous study of severe AS by Lindqvist et al. septal annular S′ increased from 5.8 cm/s before TAVI to 7.0 cm/s 1 week after TAVI, and remained largely unchanged 6 months after TAVI (7.2 cm/s) [7]. In our patient we observed a similar response at 6-weeks follow-up.

Both Tissue Doppler S′ and peak LVOT velocities are easy to measure during routine echocardiography. Tissue Doppler S′ is a robust marker of systolic LV function, particularly in the longitudinal axis, and correlates well with strain and markers of myocardial fibrosis such as late gadolinium enhancement on cardiac MR [8]. Reduced long-axis function, as reflected by S′ is common in AS patients who are often older (> 65 years) and have concomitant comorbidities such as hypertension and increased arterial stiffness [9]. However, a significant increase in S′ during DSE in AS patients with LV dysfunction may suggest that myocardial damage may be reversible and LV function can be restored following AVR, which was evident in our patient. Furthermore, although transaortic flow remained borderline (35 ml/m^2^) immediately after TAVI, it further increased to 55 ml/m^2^ at 6-weeks follow-up, which indicates early reverse LV remodeling. Mitral annular S′ may be a better immediate marker of LV recovery after pressure unloading compared with SVi. However, this is our experience from this particular case report and we therefore suggest that the clinical significance and prognostic value of mean change in mitral annular S′ and peak LVOT velocities from rest to peak stress in patients with low flow low gradient AS should be explored in future prospective studies.

This case report demonstrates that mitral annular S′ and peak LVOT velocities are reliable markers of LV intrinsic contractile function, correlate well with the indices of transaortic flow (SVi and flow rate) and may be true markers of LV recovery after unloading AS by TAVI.

We suggest the incorporation of tissue Doppler S´ and LVOT velocities into routine low-dose DSE.

## Data Availability

Data are available by the corresponding author upon reasonable request.

## Abbreviations

AS: Aortic stenosis
CT: Computed tomography
DSE: Dobutamine stress echocardiography
LV: Left ventricle
LVEF: Left ventricular ejection fraction
LVOT: Left ventricular outflow tract
NYHA: New York heart association
OD: Once daily
SVi: Stroke volume index
TAVI: Transcatheter aortic valve implantation

## References

[1] Baumgartner H, Hung J, Bermejo J, Chambers JB, Edvardsen T, Goldstein S (2017). Recommendations on the echocardiographic assessment of aortic valve stenosis: a focused update from the European Association of Cardiovascular Imaging and the American Society of Echocardiography. J Am Soc Echocardiogr.

[2] Monin JL, Quere JP, Monchi M, Petit H, Baleynaud S, Chauvel C, Pop C, Ohlmann P, Lelguen C, Dehant P, Tribouilloy C, Guéret P (2003). Low gradient aortic stenosis: operative risk stratification and predictors for long-term outcome: a multicenter study using dobutamine stress hemodynamics. Circulation..

[3] Levy F, Laurent M, Monin JL, Maillet JM, Pasquet A, Le Tourneau T, Petit-Eisenmann H, Gori M, Jobic Y, Bauer F, Chauvel C, Leguerrier A, Tribouilloy C (2008). Aortic valve replacement for low-flow/low-gradient aortic stenosis operative risk stratification and long-term outcome: a European multicenter study. J Am Coll Cardiol.

[4] Chambers JB, Rajani R, Parkin D, Saeed S (2019). Rapid early rise in heart rate on treadmill exercise in patients with asymptomatic moderate or severe aortic stenosis: a new prognostic marker?. Open Heart.

[5] Strange G, Stewart S, Celermajer D, Prior D, Scalia GM, Marwick T, Ilton M, Joseph M, Codde J (2019). Playford D; National Echocardiography Database of Australia contributing sites. Poor long-term survival in patients with moderate aortic stenosis. J Am Coll Cardiol.

[6] Saeed S, Saeed N, Grigoryan K, Chowienczyk P, Chambers JB, Rajani R (2020). Determinants and clinical significance of aortic stiffness in patients with moderate or severe aortic stenosis. Int J Cardiol.

[7] Lindqvist P, Bajraktari G, Molle R, Palmerini E, Holmgren A, Mondillo S (2010). Valve replacement for aortic stenosis normalizes subendocardial function in patients with normal ejection fraction. Eur J Echocardiogr.

[8] Saeed S, Dweck MR, Chambers J (2020). Sex differences in aortic stenosis: from pathophysiology to treatment. Expert Rev Cardiovasc Ther.

[9] Saeed S, Scalise F, Chambers JB, Mancia G (2020). Hypertension in aortic stenosis: a focused review and recommendations for clinical practice. J Hypertens.

